# *Aspergillus* Outbreak in an Intensive Care Unit: Source Analysis with Whole Genome Sequencing and Short Tandem Repeats

**DOI:** 10.3390/jof10010051

**Published:** 2024-01-06

**Authors:** Stephan J. P. Hiel, Amber C. A. Hendriks, Jos J. A. Eijkenboom, Thijs Bosch, Jordy P. M. Coolen, Willem J. G. Melchers, Paul Anröchte, Simone M. T. Camps, Paul E. Verweij, Jianhua Zhang, Laura van Dommelen

**Affiliations:** 1 Department of Intensive Care, Máxima Medical Centre, De Run 4600, 5504 DB Veldhoven, The Netherlands; 2 Centre for Infectious Disease Control, National Institute for Public Health and the Environment, Antonie van Leeuwenhoeklaan 9, 3721 MA Bilthoven, The Netherlands; 3 Department of Medical Microbiology, Radboud University Medical Centre, Geert Grooteplein Zuid 10, 6525 GA Nijmegen, The Netherlands; 4 Department of Infection Prevention and Control, Máxima Medical Centre, De Run 4600, 5504 DB Veldhoven, The Netherlands; 5 Stichting PAMM, Laboratory of Medical Microbiology, De Run 6250, 5504 DL Veldhoven, The Netherlands

**Keywords:** *Aspergillus fumigatus*, construction work, source analysis, whole genome sequencing

## Abstract

Whole genome sequencing (WGS) is widely used for outbreak analysis of bacteriology and virology but is scarcely used in mycology. Here, we used WGS for genotyping *Aspergillus fumigatus* isolates from a potential *Aspergillus* outbreak in an intensive care unit (ICU) during construction work. After detecting the outbreak, fungal cultures were performed on all surveillance and/or patient respiratory samples. Environmental samples were obtained throughout the ICU. WGS was performed on 30 isolates, of which six patient samples and four environmental samples were related to the outbreak, and twenty samples were unrelated, using the Illumina NextSeq 550. A SNP-based phylogenetic tree was created from outbreak samples and unrelated samples. Comparative analysis (WGS and short tandem repeats (STRs), microsatellite loci analysis) showed that none of the strains were related to each other. The lack of genetic similarity suggests the accumulation of *Aspergillus* spores in the hospital environment, rather than a single source that supported growth and reproduction of *Aspergillus fumigatus*. This supports the hypothesis that the *Aspergillus* outbreak was likely caused by release of *Aspergillus fumigatus* spores during construction work. Indeed, no new *Aspergillus* cases were observed in the ICU after cessation of construction. This study demonstrates that WGS is a suitable technique for examining inter-strain relatedness of *Aspergillus fumigatus* in the setting of an outbreak investigation.

## 1. Introduction

*Aspergillus* spp. is a ubiquitous saprotrophic pathogenic fungus that can be found in air, soil, and organic matter and is mainly transmitted through inhalation of spores [1]. The clinically most relevant species are *A. fumigatus*, *A. flavus*, *A. niger*, and *A. terreus*, which frequently cause invasive diseases [2,3,4]. Invasive aspergillosis (IA) is an opportunistic infection that mainly affects immunocompromised patients, such as those with hematological or oncological malignancies receiving chemotherapy [5]. However, immunocompetent patients who are critically ill or have severe viral respiratory infections are also at risk of developing IA [6]. Unfortunately, despite antifungal treatment, the mortality of IA remains high, with mortality rates ranging from 50 to 90% [7,8,9,10,11,12]. Therefore, in the case of an *Aspergillus* outbreak in a hospital setting, it is important to determine the cause of the outbreak to prevent further transmission and subsequent infections.

To identify the source of an outbreak, it can be useful to analyze the fungal strains for isogeneity. Beside standard patient samples, isolates can be taken from different sources, such as beds and blankets, walls, medical devices, and air treatment installations, to evaluate environmental involvement [13]. Thereafter, isogeneity of strains can be analyzed with several typing techniques such as random amplified polymorphic DNA (RAPD) and short tandem repeats (STRs). More recently, whole genome sequencing (WGS) has also become more accessible to type fungi [14,15]. Using WGS, isolates can be compared with a higher discriminative power in comparison to traditional techniques. To date, STR has been the primary method for typing *Aspergillus* strains in outbreak settings [14,16,17]. WGS for strain typing in outbreak analysis is already widely used in bacteriology and virology but is scarcely used in mycology [15]. Reports in which WGS was utilized in outbreak analysis have been limited to cases in which all isolates originated from a single clonal source [18,19,20].

In this report, we describe an *Aspergillus* outbreak in an intensive care unit (ICU) of a Dutch hospital. The rise in *Aspergillus*-positive cultures, mostly of *A. fumigatus*, coincided with the start of construction work, i.e., electricity maintenance, on the corridor ceiling of the ICU. Construction work in or around hospitals can increase spore release and has also been previously associated with outbreaks of IA cases [21,22,23]. After cessation of the construction work and cleaning and disinfection of the ICU, no new cases of IA or colonization were identified. Thus, it was hypothesized that the construction work could be the cause of the outbreak. Recently, WGS was used to examine the genetic diversity in *A. fumigatus* isolates and was found to be a suitable technique for examining inter-strain relatedness [24]. Furthermore, WGS has been used to investigate an outbreak of *A. fumigatus* in parrots [25]. However, to date, the use of WGS in identifying a presumed environmental source of a nosocomial outbreak has not yet been investigated. We therefore investigated the clinical utility of WGS for examining inter-strain relatedness of *A. fumigatus* strains in an outbreak setting.

## 2. Description of the Outbreak

**Figure 1:**
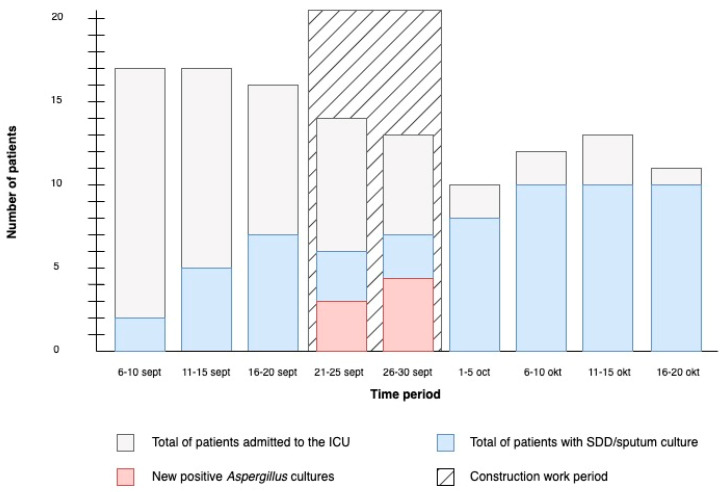
Timeline of the outbreak. Number of patients admitted to the ICU and number of positive *A. fumigatus* cultures in the ICU before, during, and after the construction work period. Construction work started on 21 September 2020 and ended on 30 September 2020. Only the first culture of each patient is included in the figure.

The construction work on the corridor of our ICU started on 21 September 2020. Following the start of construction, several new positive cultures for *Aspergillus* species emerged. Consequently, the construction work was ceased on 30 September 2020. The timeline of the outbreak is shown in Figure 1. During the outbreak, a total of nine patients had selective digestive decontamination (SDD) cultures taken, six of which had positive cultures for *Aspergillus,* and one had a positive galactomannan in bronchoalveolar lavage fluid, but remarkably did not have a positive culture for *Aspergillus*. The clinical characteristics of these patients are summarized in Table 1. Two patients were diagnosed with probable COVID-19-associated pulmonary aspergillosis (CAPA) following the 2020 ECMM/ISHAM criteria, for which they received antifungal treatment [26]. One of these patients eventually died following refractory sepsis. Another patient was initially admitted to the ICU due to neutropenic septic shock and was later diagnosed with probable IA following the guidelines of the European Organization for Research and Treatment of Cancer/Mycoses Study Group Education and Research Consortium (EORTC/MSGERC) [27]. Eventually, the patient died due to sepsis and pneumonia. Lastly, four patients had colonization with *A. fumigatus* and/or *A. flavus*. After cessation of construction work and cleaning and disinfection of the ICU, no newly colonized or infected patients were identified.

## 3. Materials and Methods

### 3.1. Clinical Sample Collection

According to the standard practice in the ICU of our hospital, microbiological, including fungal, surveillance cultures in the context of SDD were collected twice a week from patients with an (expected) ICU stay of at least 3 days and/or receiving mechanical ventilation for at least 2 days.

After the outbreak was thought to be linked to the construction work, a Sabouraud Dextrose agar (SDA) (CM0041) was added to all new respiratory (sputum and throat swab) samples immediately after cessation of construction work, to enhance the recovery of fungi. Respiratory and surveillance samples that had been taken prior to discovery of the outbreak, but were still being processed in the microbiology laboratory, were also subcultured on SDA. We stopped using SDA for the respiratory samples after no new cases were identified at mid-October.

### 3.2. Environmental Investigation

Immediately following cessation of construction work and the cleaning and disinfection of the ICU, an environmental survey was conducted. This consisted of the monitoring of air quality inside the ICU, from the 30 September until the 5 October 2020. To this end, an external company took environmental samples from seven unoccupied ICU patient rooms and the ICU corridor. Additionally, environmental samples were taken from two randomly selected patient rooms on a regular inpatient ward to use as control samples. The samples were collected with the Solair 3100 (airborne particle count), ActiveCount 100H (microbial samples), and ActiveCount 100 (microbial samples) (Lighthouse Worldwide Solutions, Boven-Leeuwen, The Netherlands). According to standard practice for environmental sampling, 1000 L of air was used per sample. Airborne particles with a size equal to or greater than 0.5 μm were measured and subjected to analysis.

All particle counts were performed in duplicate and further calculations were performed using the average of the two results. For cultivation and isolation of the fungi from the air environmental samples, defined as colony forming units per m^3^ (CFU p/m^3^), the Sabouraud Dextrose agar (CM0041) and Tryptone Soya Agar (CM0131) (Oxoid Limited, ThermoFisher Scientific, Hampshire, UK) plates were used.

### 3.3. Whole Genome Sequencing and Genotyping

#### 3.3.1. Isolates Culturing and DNA Isolation

A single colony culture was initiated by transferring a small amount of cultured *Aspergillus fumigatus* and suspending it in 0.5% Tween-20 in saline. Subsequently, 1 µL of this suspension was spread onto Sabouraud agar (ThermoFisher cat.nr. CM0041B). Following an incubation period at 37 °C, a singular colony was selected and suspended in 0.5% Tween-20 in saline. This suspension was used to inoculate a slanted Sabouraud agar tube with a 10 µL loop and then incubated at 37 °C until it reached sufficient growth. Isolation was considered complete when a dense layer of gray/green conidia covered the agar surface.

DNA was isolated from conidia using a phenol-chloroform extraction method followed by precipitation. A spatula tip of glass beads (0.4–0.6 mm diameter) was put in a 2.0 mL safelock tube, followed by the addition of 700 µL breaking buffer [28].

As much conidia as possible was collected from slanted agar tubes with a cotton swab and resuspended in the 2.0 mL safelock tubes with glass beads and breaking buffer. These tubes were vortexed for 30 s and incubated at 60 °C in a shaking heating block set at maximum shaking speed (1600 rounds per minute (RPM)). Following incubation, 700 µL of phenol-chloroform-isoamylalcohol (25:24:1) was added to the suspension, shaken vigorously for 1 min, and subsequently centrifuged at 4 °C for 10 min at 10,000× *g*. From this, 600 µL of the upper phase was carefully transferred to a new 1.5 mL safelock tube. Next, 600 µL of chloroform-isoamylalcohol (49:1) was added, shaken for 1 min, and centrifuged at 4 °C for 10 min at 10,000× *g*. Following this step, 400 µL of the upper phase was transferred to another clean 1.5 mL safelock tube.

To precipitate the DNA, 40 µL of 3 M sodium acetate and 400 µL of ice-cold 80% 2-propanol (−20 °C) were added, and the tube was inverted to mix the contents. The tubes were then incubated overnight at −20 °C, followed by centrifugation at −9 °C for 15 min at 10,000× *g*. The supernatant was removed, and the DNA pellets were washed with 250 µL of ice-cold 80% ethanol (−20 °C). Subsequently, the DNA pellet was dissolved in 50 µL of DEPC-treated H_2_O and incubated for 15 min at 60 °C [28].

#### 3.3.2. Whole Genome Sequencing

For the preparation of sequencing libraries, the Illumina DNA prep kit (Illumina, San Diego, CA, USA) was used according to the manufacturer’s protocol. Sequencing was performed in a paired-end 2 × 150 bp mode on an Illumina NextSeq 550 system (Illumina, San Diego, CA, USA).

#### 3.3.3. STR Typing

As a control, we also performed STR typing as described previously [17]. The STR markers used in this study are a subset of the panel, involving the M2 multiplex (containing the STR*Af*-2A, STR*Af*-2B, and STR*Af*-2C dinucleotide repeat markers), M3 multiplex (containing the STR*Af*-3A, STR*Af*-3B, and STR*Af*-3C trinucleotide repeat markers), and M4 multiplex (containing the STR*Af*-4A and STR*Af*-4B tetranucleotide repeat markers). In each multiplex reaction, different fluorescent labels were used to discriminate among the individual markers. Repeat numbers at each locus were determined by PCR and subsequent sequencing.

#### 3.3.4. Unrelated Sequencing Data Acquisition

WGS analysis of patient isolates and environmental isolates unrelated to the outbreak was used for comparative analysis. We added 11 isogenic isolates from a study by Ballard et al. [29] and two isolates from a study of Engel et al. [30] as unrelated isolates. We also added an isolate from *A. fischeri* as an outlier for the phylogenetic analysis. Fastq files of the study of Ballard et al. and the *A. fischeri* isolate were downloaded from The Sequence Read Archive using SRA-tools [31,32]. The fastq files from the study by Engel et al. were provided to us by the authors.

#### 3.3.5. Data Analysis

A part of the RIVM in-house Juno-Assembly pipeline [33] was used to trim the raw reads of all samples using Trimmomatic v. 0.38 [34] with a window size of 5 and a Phred score of 28 as average required quality. The quality of the reads was assessed with FastQC v. 0.11.8 [35]. To determine the average coverage, the cleaned reads were used for a de novo assembly using SPAdes 3.14.0 (k: (21,33,55,77,99)), and BBmap 38.22 was used to determine the alignment metrics of the resulting scaffolds [36,37].

The Split Kmer Analysis toolkit v. 1.0 (SKA) [38] was used to determine the genetic distance between samples based on k-mers without using a reference genome. From this toolkit, ska fastq with a coverage cut-off of 20 k-mers, a k-mer length of 15, and a minimum allowable minor allele frequency of 0.5 was used to create k-mer files per sample. In addition, ska align was used to align the k-mer files. In this analysis, only variant sites were included, and a minimum proportion of isolates required to possess a split k-mer parameter of 1 in an analysis without an outlier and a value of 0.96 in an analysis with outlier was used. To determine the Single Nucleotide Polymorphism (SNP) distances between the samples based on the alignment, Snp-dists v. 0.7.0 [39] was used. A phylogenetic tree was created using IQ-TREE v. 1.6.12 using UFBoot2 [40] 1000 and ModelFinder [41], and visualized using iTOL v. 6.1.2 [42].

## 4. Results

### 4.1. Whole Genome Sequencing and Microsatellite Typing

A total of 30 isolates were included for the WGS analysis, of which six isolates were from patients admitted to the ICU during the outbreak, two were from patients admitted to the ICU before the outbreak, and four were nosocomial environmental isolates taken after the outbreak. For comparative analysis and context, four epidemiological unrelated isolates collected from patients several years before the outbreak, eleven isogenic isolates from a study by Ballard et al. [29], which all came from a patient with severe chronic obstructive pulmonary disease, two isogenic samples from a study by Engel et al. [30] derived from a cystic fibrosis (CF) patient, and *Aspergillus fischeri* NRRL 4585 (GenBank accession: JAAKEP000000000) as an outlier were also included (Table 2).

**Figure 2:**
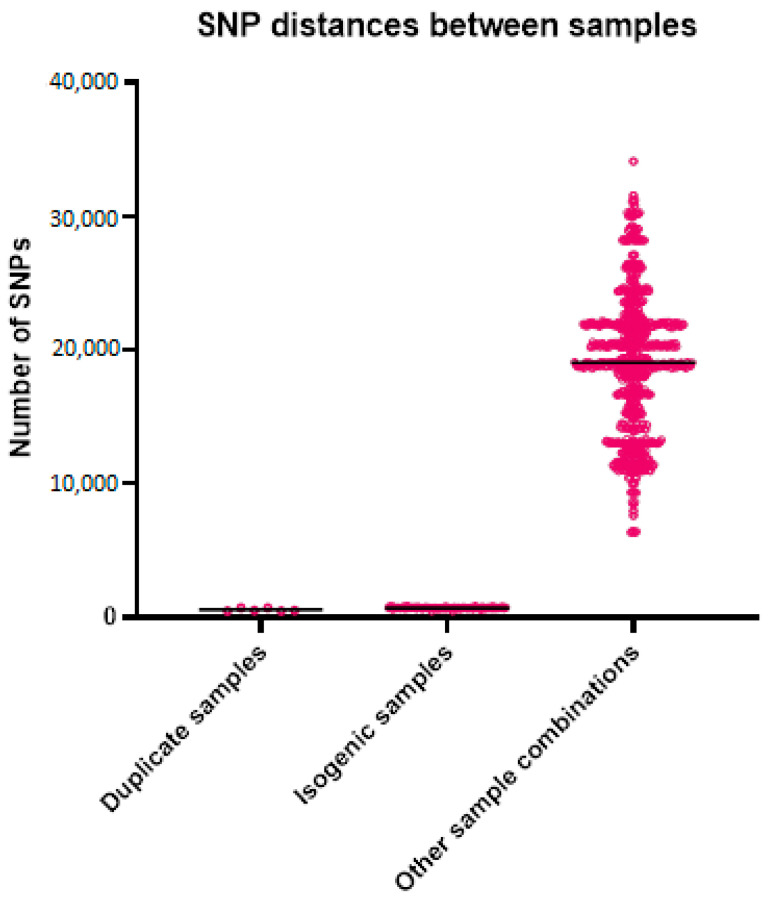
Genetic distances between the different sample groups. Each point in the graph represents the genetic distance between two samples.

**Figure 3:**
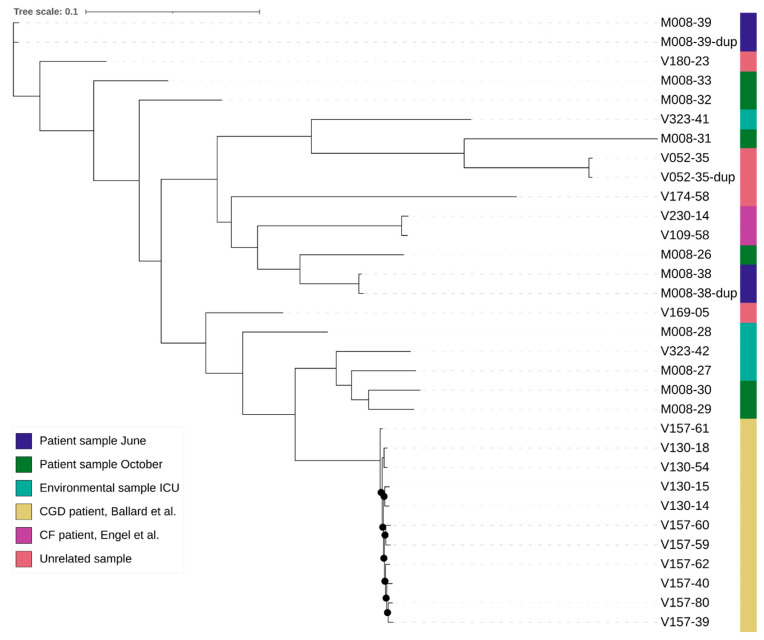
Phylogenetic tree of the outbreak related samples, isogenic patient samples, and unrelated samples [29,30]. Branches with UFBoot bootstrap values >90% are indicated with a black dot. The tree of the analysis including the outlier can be found in the Supplementary Files.

For the WGS, the average coverage was 149 reads for all samples, with 52 for the lowest coverage and 288 for the sample with the highest coverage. The number of k-mers per sample varied from 25,031,349 to 31,271,726, with an average of 27,274,922, which roughly corresponds to the length of the genomes. The alignment length for the analysis with the outlier was 76,755 bases and without the outlier was 229,806 bases. The mean SNP distance based on the k-mer alignment without the outlier was 696 between the isogenic strains, 563 between the duplicates, and 19,055 between all other samples (Table 3 and Figure 2). The best-fit model according to ModelFinder was SYM+ASC+R2 for the analysis with the outlier and TVM+F+ASC+R3 without the outlier. Based on the alignment and these models, two phylogenetic trees were created (Figure 3). Both trees show that the isogenic strains and the duplicate isolates cluster together. The isolates related to the outbreak are scattered throughout the tree, with the strains from the studies and the unrelated strains in between. None of the isolates related to the outbreak cluster together. The STR results are shown in Table 4. None of the isolates from the outbreak have an identical profile. Only the profiles of the duplicates are the same or differ by a maximum of one repeat.

### 4.2. Environmental Investigation

The first day after cessation of construction work, the number of particles of ≥0.5 μm in the ICU corridor was 46.7 × 10^5^ particles per m^3^. Two days after cessation, this decreased to 2.0 × 10^5^ particles per m^3^. Airborne particle analysis from the first day after cessation of construction work was available for two ICU patient rooms, indicating 15.6 × 10^5^ particles per m^3^ and 7.8 × 10^5^ particles per m^3^, respectively. Two days after cessation, particles were taken from seven ICU patient rooms with a median number of particles per m^3^ of 1.1 × 10^5^ (range 0.8 × 10^5^–1.4 × 10^5^). In comparison, in the regular inpatient ward, the median number of particles of ≥0.5 μm in the two patient rooms was 2.2 × 10^5^ particles per m^3^ (range 2.1 × 10^5^–2.3 × 10^5^). The environmental samples taken from the ICU patient rooms, the ICU corridor, and the patient rooms in the regular inpatient ward showed 0–2 CFU/1000 L for *Aspergillus fumigatus*, which was considered acceptable.

## 5. Discussion

In this report we used WGS and STR to analyze the source of a nosocomial *Aspergillus* outbreak. We demonstrate that WGS is a suitable technique for examining inter-strain relatedness of *A. fumigatus* in outbreak analysis. The WGS analysis of the patient respiratory samples and the environmental samples showed that none of the strains were related to each other. The most similar isolates differed by more than 6000 SNPs and the most unrelated strains differed by more than 34,000 SNPs. Control STR analysis confirmed the lack of inter-relatedness of the samples. The lack of genetic similarity of isolates suggests an accumulation of *Aspergillus* spores in the hospital environment, rather than a single clonal source that supported growth and reproduction of *A. fumigatus,* as we would then expect more strains to be related to each other. Most previous studies investigating nosocomial *A. fumigatus* outbreaks were not able to link environmental genotypes to those recovered from patients [21,22,43]. This may be due to the limited number of *A. fumigatus* isolates that are generally recovered from the hospital environment or the timing of environmental sampling, which usually takes place only after an outbreak has been detected. Although *A. fumigatus* creates clonal progeny when undergoing asexual reproduction, it is likely that active growth (and reproduction) does not take place in the hospital environment [44]. A more likely explanation is that, over time, genetically different spores accumulate in certain areas (e.g., ceiling spaces) and may be released during construction activities. Our genotyping data support such a hypothesis, which is also further supported by the absence of new cases of *Aspergillus* once the construction activities were ceased.

An advantage of using WGS over STR in fungal genotyping is that not only can the epidemiology of the isolates be examined with a greater discriminatory power at a comparatively low labor and monetary expense, but also the resistance and virulence of the isolates can be mapped [45,46]. On the other hand, the disadvantage of WGS is that the SNP difference between two isolates can differ depending on the method, parameters, and set of isolates used. Therefore, developing a whole genome multi-locus sequence typing (wgMLST) method may be a better way to monitor the epidemiology of *A. fumigatus*. This method allows each laboratory to independently sequence and analyze its isolates, using a consistent set of conserved loci, and then aggregate the results. It is more sensitive than STR and it is more interchangeable than SNP typing.

To further explore the presumed environmental source of the outbreak, we also performed an environmental investigation. To date, there are no available guidelines concerning airborne particles and CFU from environmental samples for nursing wards and ICUs with which to compare our results. Therefore, to be able to give more insight into our data, we compared them to the ISO cleanroom standards. The ISO norm 7, classification of air cleanliness in terms of concentration of airborne particles in an operating theatre class 1 and 2 (NEN EN ISO 14644-1), is the most appropriate ISO norm for our ICU and prescribes a maximum number of ≥0.5 μm particles of 3.5 × 10^5^ particles per m^3^ [47]. We found that the environmental samples taken from the ICU corridor and ICU patient rooms one day after cessation of construction work showed particle counts that largely exceeded that threshold. However, on the second day following cessation of construction work, there was a notable decrease in particle counts, now falling below the 3.5 × 10^5^ particles per m^3^ threshold. This highlights that timely air quality monitoring could be an important measure in preventing similar outbreaks. Lastly, environmental samples showed no more than two fungal CFU/1000 L, but no cut-off exists for this measurement.

Following the cessation of construction work and the cleaning and disinfection of the ICU, no new cases of IA or colonization were seen. The environmental samples (for microbiological sampling and particle count) resembled those taken in the control patient rooms on the regular nursing ward. These findings underscore that the cessation of construction work, and the cleaning and disinfection of the patient rooms was the appropriate intervention to prevent further spread of the outbreak.

The primary limitation of this study was the limited number of patient and environmental samples that were available for analysis. Because of this, our study most likely lacked the power to identify identical strains between the patient samples and the environmental samples. Nonetheless, our conclusion remains the same. Another limitation is the absence of environmental sampling data from during the construction work period, as the construction work was immediately ceased once it was suspected to be the source of the *Aspergillus* outbreak. To enhance future analyses and gain a more comprehensive understanding of environmental pathogens during construction work in healthcare setting, we would recommend collecting environmental samples before, during, and after construction work.

In conclusion, this study found WGS to be a suitable technique for examining inter-strain relatedness of *A. fumigatus* for source analysis in the setting of a nosocomial outbreak investigation. Our findings suggest that the *Aspergillus* outbreak in our ICU was likely caused by construction work rather than a single source. Therefore, in case of construction work that is carried out in the proximity of patient rooms, it is important to not only adhere to standard infection control protocols, such as enclosing the area where the work is taking place to prevent or minimize dust dispersion, but to also consider additional measures, such as timely routine monitoring of air quality. This comprehensive approach could contribute to a more robust strategy for preventing and managing nosocomial outbreaks associated with environmental factors.

## Figures and Tables

**Table 1:** Characteristics of patients with probable invasive aspergillosis or colonization with *A. fumigatus* or *A. flavus*. SDD = Selective digestive decontamination; NA = Not available; GMI = Galactomannan index (≥0.5 = positive).

	Patients						
Characteristic	1	2	3	4	5	6	7
Age, years	76	88	73	71	69	76	73
Sex	Male	Male	Female	Female	Female	Male	Male
Clinical risk factors for IA	None	None	Prolonged cortico-steroid use	None	None	Prolonged cortico-steroid use	Neutro-penia due to urothelial-carcinoma
Reason for ICU admission	COVID-19	Post-operative care	COVID-19	Septic shock	COVID-19	COVID-19	Septic shock
SDD culture for *Aspergillus* spp.	*flavus*	*fumigatus + flavus*	*fumigatus*	*fumigatus*	*fumigatus*	*fumigatus + flavus*	NA
Bronchoalveolar lavage							
Fungal culture	Negative	NA	Negative	NA	Positive	Positive	Negative
Galactomannan (GMI)	Negative	NA	Negative	NA	Positive (4.33)	Positive (4.32)	Positive (3.73)
Diagnosis IA	No, colonization	No, colonization	No, colonization	No, colonization	Yes, probable CAPA	Yes, probable CAPA	Yes, probable IA
Outcome	Discharged from ICU	Discharged from ICU	Discharged from ICU	Discharged from ICU	Discharged from ICU	Deceased	Deceased

**Table 2:** The samples included in the WGS analysis, including the origin and period of sample collection. * = samples unrelated to the outbreak that were included in duplicate to better understand technical errors.

Sample Name	Origin	Time of Sampling
V109-58	CF patient, Engel et al. [30]	2010
V230-14	CF patient, Engel et al. [30]	2017
V130-14	CGD patient, Ballard et al. [29]	2011
V130-15	CGD patient, Ballard et al. [29]	2011
V130-18	CGD patient, Ballard et al. [29]	2011
V130-54	CGD patient, Ballard et al. [29]	2011
V157-39	CGD patient, Ballard et al. [29]	2013
V157-40	CGD patient, Ballard et al. [29]	2013
V157-59	CGD patient, Ballard et al. [29]	2013
V157-60	CGD patient, Ballard et al. [29]	2013
V157-61	CGD patient, Ballard et al. [29]	2013
V157-62	CGD patient, Ballard et al. [29]	2013
V157-80	CGD patient, Ballard et al. [29]	2013
M008-27	Environmental sample ICU	October 2020
M008-28	Environmental sample ICU	October 2020
V323-41	Environmental sample ICU	October 2020
V323-42	Environmental sample ICU	October 2020
NRRL-4585	Outlier	does not apply
M008-38 *	Patient sample June	June 2020
M008-39 *	Patient sample June	June 2020
M008-26	Sample October patient 1	October 2020
M008-29	Sample October patient 2	October 2020
M008-30	Sample October patient 3	October 2020
M008-31	Sample October patient 4	October 2020
M008-32	Sample October patient 5	October 2020
M008-33	Sample October patient 6	October 2020
V052-35 *	Unrelated patient sample	2006
V169-05	Unrelated patient sample	2014
V174-58	Unrelated patient sample	2015
V180-23	Unrelated patient sample	2015

**Table 3:** Descriptive statistics of the SNP distances of the sample groups.

	Duplicate Samples	Isogenic Samples	Other Sample Combinations
Number of values	6	112	874
Minimum	469	451	6330
Maximum	705	863	34,104
Range	236	412	27,774
Mean	563	696	19,055
Std. Deviation	112	64	5244
Std. Error of Mean	46	6.0	177

**Table 4:** Results of loci STR2, STR3, and STR4 of the isolates related to the outbreak, and two duplicates.

Sample	STR2			STR3			STR4		
	A	B	C	A	B	C	A	B	C
M008-38	22	18	15	102	11	17	21	10	9
M008-38-dup	22	18	15	102	11	16	21	10	9
M008-39	24	18	12	52	7	11	9	8	5
M008-39-dup	24	18	12	52	7	11	9	8	5
M008-30	17	27	4	28	9	20	6	7	5
M008-28	28	16	11	28	10	20	6	7	6
M008-27	17	10	5	37	9	27	7	7	9
M008-26	22	18	17	27	19	16	8	16	7
M008-33	17	24	11	25	10	8	25	8	7
M008-29	10	10	5	29	20	19	7	6	5
M008-31	13	19	8	25	8	9	6	8	25
M008-32	24	21	13	22	8	26	8	8	5

## Data Availability

Publicly available datasets were analyzed in this study. These data can be found here: https://github.com (accessed on 1 November 2023).

## Supplementary Materials

The following supporting information can be downloaded at: www.mdpi.com/article/10.3390/jof10010051/s1, Figure S1: Phylogenetic tree of the outbreak related samples, isogenic patient samples, and unrelated samples (including the outlier).
