# Supplementary figures and images for: Aspergillus Outbreak in an Intensive Care Unit: Source Analysis with Whole Genome Sequencing and Short Tandem Repeats
Source: J Fungi (Basel). 2024 Jan 6;10(1):51. doi: 10.3390/jof10010051 (PMC10817286; doi:10.3390/jof10010051)

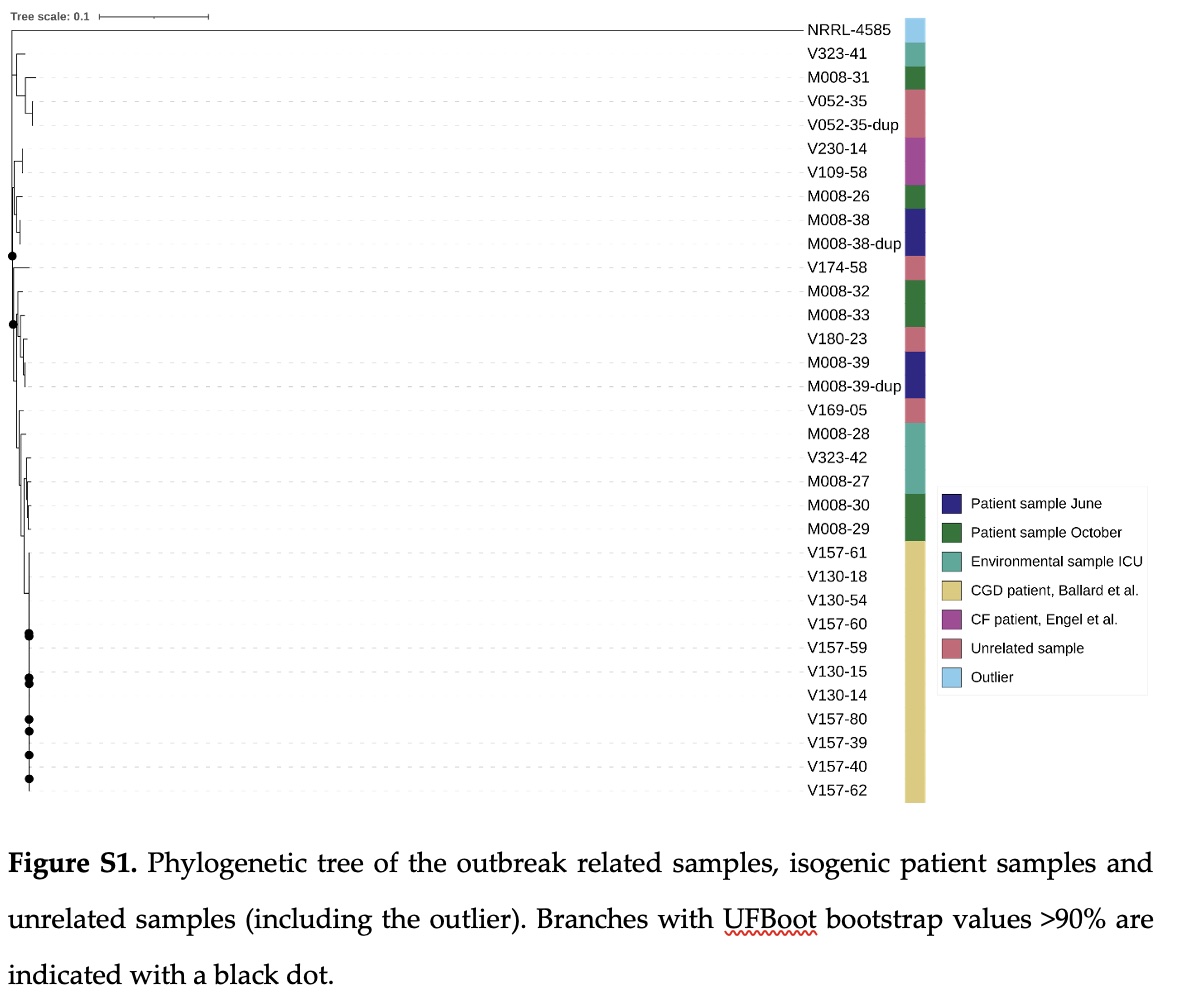

Supplement: Supplementary file 1 [file jof-10-00051-s001.zip › Figure S1.jpg]
